# Lipopolysaccharide-induced active telocyte exosomes alleviate lipopolysaccharide-induced vascular barrier disruption and acute lung injury via the activation of the miRNA-146a-5p/caspase-3 signaling pathway in endothelial cells

**DOI:** 10.1093/burnst/tkae074

**Published:** 2025-01-14

**Authors:** Xiaoqin Huang, Haoran Zhang, Yuhong Luo, Xin Yi, Zengding Zhou, Feng Guo, Lei Yi

**Affiliations:** Department of Burn, Ruijin Hospital, Shanghai Jiao Tong University School of Medicine, 197 Second Ruijin Road, Huangpu District, Shanghai, 200025, China; Department of Orthopedics, The Second Affiliated Hospital of Harbin Medical University, 246 Xuefu Road, Nangang District, Harbin, 150001, Heilongjiang, China; Department of Burn, Ruijin Hospital, Shanghai Jiao Tong University School of Medicine, 197 Second Ruijin Road, Huangpu District, Shanghai, 200025, China; Department of Burn, Ruijin Hospital, Shanghai Jiao Tong University School of Medicine, 197 Second Ruijin Road, Huangpu District, Shanghai, 200025, China; Department of Burn, Ruijin Hospital, Shanghai Jiao Tong University School of Medicine, 197 Second Ruijin Road, Huangpu District, Shanghai, 200025, China; Department of Plastic Surgery, Shanghai Jiaotong University Affiliated Sixth People’s Hospital, 600 Yishan Road, Xuhui District, Shanghai, 200235, China; Department of Burn, Ruijin Hospital, Shanghai Jiao Tong University School of Medicine, 197 Second Ruijin Road, Huangpu District, Shanghai, 200025, China

**Keywords:** Telocyte, Endothelial cells, Acute lung injury, Sepsis, miRNA-146, Organ dysfunction syndrome, Lipopolysaccharide, LPS

## Abstract

**Background:**

Lipopolysaccharide (LPS)-induced apoptosis of lung microvascular endothelial cells (ECs) is the main reason of lung edema and acute lung injury (ALI) in septic conditions. Telocytes (TCs) are a distinct type of interstitial cells found around the lung microvasculature, which may protect ECs through the release of shed vesicles. However, whether TCs protect against LPS-induced EC apoptosis and ALI has not been determined.

**Methods:**

The protective effects of TCs on ECs were assessed *in vitro* using transwell assays and flow cytometry, and *in vivo* using an LPS-induced mouse ALI model. RNA sequencing was used to identify miRNA-146a-5p as a key component of TC-derived exosomes. The functions of miRNA-146a-5p were further evaluated by western blotting, flow cytometry, and transendothelial electrical resistance measurements.

**Results:**

We demonstrated that LPS stimulation induced the secretion of active exosomes from TCs, which inhibited LPS-mediated apoptosis of ECs and reduced ALI in mice. Moreover, miRNA-146a-5p was identified as the main bioactive molecule in TC-derived exosomes, capable of inhibiting LPS-induced caspase-3 activation and apoptosis in ECs.

**Conclusions:**

Our results indicate that TCs effectively prevent LPS-induced EC apoptosis and ALI through the release of exosomes, with subsequent activation of the miRNA-146a-5p/caspase-3 signaling pathway in ECs.

Highlights

- Telocytes surround the pulmonary microvascular endothelial cells and play a very important protective role under sepsis condition.
- LPS promotes telocytes to synthesize and secrete active exosomes containing a large number of active molecules, which inhibits the LPS-induced pulmonary microvascular endothelial cells injury.
- MiRNA-146a-5p, which is a key active factor in the LPS-induced active exosomes in telocytes, alleviates LPS-induced endothelial cell apoptosis by inhibiting the caspase-3 signaling pathway.

## Background

Sepsis is a life-threatening organ dysfunction syndrome caused by a dysregulated host response to infection [1, 2]. Among septic patients admitted to ICUs worldwide, the most common source of infection is the lungs (64%), and more than half (62%) of the microorganisms isolated are gram-negative bacteria [1]. Lipopolysaccharide (LPS) is the main component of the outer surface of gram-negative bacteria. *In vitro* experiments have shown that LPS can rapidly induce the apoptosis of endothelial cells (ECs) [3–7], which further compromises the integrity of the lung alveolar–capillary membrane, leading to acute lung injury (ALI) [8, 9]. Although numerous studies have elucidated the molecular mechanisms and targets of LPS-induced apoptosis in lung microvascular ECs [3, 4, 10, 11], little is known about effective methods to block the LPS-induced apoptosis of lung vascular ECs and ALI in clinical practice. In recent years, increasing evidence has shown that cell–cell communication may play an important role in the protective mechanisms of lung vascular ECs [12]. However, whether protective cells around pulmonary microvessels inhibit LPS-induced lung vascular EC injury has not been reported in the literature.

As a recently defined type of mesenchymal cell, telocytes (TCs), which function in signal transduction, tissue formation, and renewal, can be found around capillaries and in most organs, including the lungs [13–16]. TCs mainly carry out cell–cell communication by secreting exosomes that transfer bioactive molecules, including proteins and nucleic acids (such as noncoding RNA), to various recipient cells, thereby protecting them [17–19]. However, it remains unclear whether TCs protect lung vascular ECs against LPS-induced apoptosis and subsequent ALI under septic conditions.

In this study, we aimed to determine whether TCs inhibit the LPS-induced apoptosis of ECs and explore the underlying molecular mechanisms of the protective effect of TCs on ECs during sepsis. We found that LPS-induced active TC exosomes effectively blocked LPS-induced EC injury and alleviated LPS-induced ALI. We also found that after LPS stimulation, the secretion and volume of active TC exosomes increased significantly, and these exosomes were then quickly absorbed by ECs and were distributed mainly in the cytoplasmic region of ECs. Importantly, in our study, we showed that miRNA-146a-5p was the key protective molecule in LPS-induced active TC exosomes and was transferred into ECs to inhibit LPS-induced caspase-3 signaling pathway activation and cellular apoptosis in ECs. Our study reveals the protective role and molecular mechanisms of TCs in ECs during sepsis, which may provide a novel therapeutic direction for lung vascular EC injury and ALI under septic conditions.

## Methods

### Animals

Male C57BL/6 mice at 8 weeks old (20 ± 2 g) were obtained from the Experimental Animal Center of Shanghai Jiao Tong University Affiliated Sixth People’s Hospital, Shanghai, China. This study was approved by the Institutional Animal Care and Use Committee of Shanghai Jiao Tong University (SYXK2016-0020). The animal feeding environment was as follows: 25°C, 12 h/12 h light/dark, 50% humidity, and free access to sterilized food and water. The mice were intratracheally instilled with 2.0 mg/kg of LPS (Sigma-Aldrich, L2630) [20] [or with phosphate buffered saline (PBS) as a control] after anesthesia by isoflurane (2%). After 12 h of the LPS stimulation, the purified exosomes (3 μg/g) were transplanted via the tail vein. For histological assessment of sepsis-induced ALI, the mouse lungs were removed and fixed in 4% paraformaldehyde. The fixed lungs were embedded in paraffin and cut into 5-μm sections. H&E staining was performed, and the slides of each group were assessed under high-power fields.

### Isolation and culture of mouse lung telocytes

Mouse lung TCs were isolated and prepared as described previously [14]. In brief, the C57BL/6 mouse lung tissues were removed, cut into small pieces, and collected into sterile tubes. The tissues were digested with 100 μg/ml liberase (Roche, 5401127001) and 40 μg/ml deoxyribonuclease I (Roche, 10104159001) to obtain a single-cell suspension. After being filtered through a 40-μm-diameter cell strainer, the dispersed cells were collected by centrifugation at 300×*g* for 7 min. Cells were resuspended in Dulbecco’s Modified Eagle Medium (Sigma-Aldrich, D6429), supplemented with 10% fetal bovine serum (FBS, Capricorn Scientific, FBS-12A), 100 IU/ml penicillin, and 100 μg/ml streptomycin, and cultured in an incubator, with 5% CO_2_ in air, at 37°C, for 30 min, to wipe off most fibroblasts. The cell suspension was then transferred to other plates and cultured for another 12 h. Culture medium was changed every 48 h. Cells were examined by an inverted live cell microscope (DMi8; Leica).

Mouse lung microvascular ECs were obtained from Zhong Qiao Xin Zhou Biotechnology Co., Ltd (PRI-MOU-00001, Shanghai, China) and maintained in Mouse lung microvascular endothelial cells complete medium (PCM-M-01) in a humidified 37°C, 5% CO_2_ incubator. The medium was changed at 48-h intervals.

### Isolation of exosomes of mouse lung telocytes

Exosomes were isolated from the culture supernatants of TCs or TCs stimulated by LPS (1 μg/ml) for 24 h, or transfected with siRNA, based on previous procedures with some modifications [17]. In brief, cultural supernatants were centrifuged at 10 000×*g* for 30 min, and cells and cellular debris were discarded. After being filtered through a 0.22-μm membrane, the supernatants were centrifuged at 120 000×*g* at 4°C for 2 h. The sediment which is the isolated exosomal pellet was washed once with sterile PBS and resuspended in 200 μl of PBS. Exosomal marker proteins were characterized by western blotting using CD9 (Abcam, ab92726) and CD81 (Cell Signaling Technology, 10037) antibodies. The number and size of exosomes were measured by nanoparticle tracking analysis (NTA) with NanoSight 500 system (NanoSight Technology, Malvern, UK). The total protein concentration of the exosomes was detected with bicinchoninic acid (BCA) protein assay kit (Yeasen Biotech, 20201ES86). Moreover, exosome morphology was determined via transmission electron microscopy (FEI Tecnai 12; Philips, Netherlands). For exosome labeling, TC exosomes were labeled with PKH67 green fluorescent membrane dye (Sigma-Aldrich, MINI67). The labeled exosomes were then incubated with human pulmonary microvascular endothelial cells (HPMECs) at 37°C, 5% CO_2_ for 3 h and measured with a fluorescence microscope.

### Confocal immunofluorescent analysis

For lung staining, mouse lungs were removed and fixed in ice-cold 4% paraformaldehyde, embedded in paraffin, and cut into 4-μm sections. The lung slides were deparaffinized and rehydrated by xylene and ethanol at different concentrations (100%, 95%, 80%, 70%, and 50%). The antigen was repaired by using a microwave in 10 nM sodium citrate buffer (pH 6.0) for 10 min. Samples were blocked with blocking solution (10% donkey serum and 0.3% Triton X-100 in PBS) at room temperature for 30 min. CD31 (Invitrogen, MA1-26196, Hamster) antibody was added to the slides and incubated at 4°C overnight. Slides were washed with PBS three times and incubated with fluorescein isothiocyanate (FITC) conjugated secondary antibody at room temperature for 1 h. TCs were incubated with CD34 (Abcam, ab8158, Rat), Vimentin (Cell Signaling Technology, 5741, Rabbit), and c-kit (R&D, AF1356, Goat) primary antibodies. After washing thrice with PBS, fluorescent-conjugated secondary antibodies were incubated with coverslips for 1 h at room temperature. All samples were stained with DAPI (D1306; Thermo Fisher Scientific) before being examined under a fluorescence microscope (Leica TCS SP8 STED, Osaka, Japan).

### Western blot assay

Cultured ECs were washed twice with cold PBS and lysed with cold radioimmunoprecipitation assay lysis buffer (Abcam, Cambridge, MA, USA) containing protease and phosphatase inhibitors (Sangon Biotech, Shanghai, China). The lysates were maintained on ice for 30 min and centrifuged at 10 000×*g* for 10 min at 4°C. The protein concentrations of the lysates were determined using a BCA protein assay. Protein samples were subjected to SDS-PAGE and electrotransferred to nitrocellulose membranes (Thermo Fisher Scientific, Waltham, MA, USA). The membranes were blocked in 5% bovine serum albumin or 5% nonfat milk in TBST (Tris-buffered saline and Tween-20) for 1 h, and then incubated with antibodies to the target proteins (caspase-3, cleaved caspase-3, and GAPDH dilutions were 1:1000) overnight at 4°C. After washing three times with TBST, the membranes were incubated with the appropriate horseradish peroxidase–conjugated secondary antibodies (1:10 000 in TBST) for 1 h at room temperature. After washing three times with TBST, the immunoreactive protein bands were visualized using an ECL detection kit (Thermo Scientific, 32106, USA) and analyzed using Quantity One software.

### Markers of apoptosis

ECs were incubated separately with PBS, LPS, and TC exosomes (derived from TCs with or without pre-stimulated by LPS, control siRNA, or miR146 siRNA), for the indicated times. A total of 5 × 10^5^ cells/ml were harvested and suspended in 500 μl binding buffer (2% FBS + 2 mM EDTA in PBS). Subsequently, cell apoptosis was quantified by staining with 5 μl of Annexin V-fluorescein isothiocyanate and 5 μl of propidium iodide (PI) for 15 min in the dark, and the results were analyzed by FlowJo software (LLC).

### RNA sequencing

RNA purity was verified using a Fragment Analyzer Bioanalyzer with a standard sensitivity RNA analysis kit (A54984; Thermo Fisher Scientific), followed by RNA-Seq. Briefly, the mRNA was fragmented after poly (A)-based mRNA enrichment with oligo (dT) magnetic beads using 18 total RNA samples as the input material. Following the first and second strands, complementary DNA synthesis, end repair, 3′-adenylation, and ligation of fork-tail linkers were performed. Following polymerase chain reaction amplification, the library quality was verified on the Fragment Analyzer Bioanalyzer, with a fragment size of 150 bp, and the paired-end library was sequenced using BGISEQ-2000 (read length: 2 × 150).

RNA sequencing could be modeled as a random sampling process, in which each read is sampled independently and uniformly from every possible nucleotide in the sample. Under this assumption, the number of reads coming from a gene (or transcript isoform) follows a binomial distribution (and could be approximated by a Poisson distribution). Using the statistical model described above, DEGseq proposes a novel method based on the MA-plot, which is a statistical analysis tool having been widely used to detect and visualize intensity-dependent ratio of microarray data. Let C1 and C2 denote the counts of reads mapped to a specific gene obtained from two samples, with Ci ~ binomial(ni,pi), i = 1,2, where ni denotes the total number of mapped reads and pi the probability of a read coming from that gene. We define M = log2C1 − log2C2, and A = (log2C1 + log2C2)/2. It can be proved that under the random sampling assumption, the conditional distribution of M given that A = a (a is an observation of A) follows an approximate normal distribution. For each gene on the MA-plot, we do the hypothesis test of H0: p1 = p2 versus H1: p1 ≠ p2. Then, a *P*-value could be assigned based on the conditional normal distribution. The *P*-values calculated for each gene are adjusted to *Q*-values for multiple testing corrections by two alternative strategies. To improve the accuracy of DEGs’ result, we defined a gene as a DEG (differentially expressed gene) when reads number foldchange ≥2 and *Q*-value ≤0.001.

### Transfection with miRNA-146a-5p siRNA *in vitro*

Stealth RNA interference duplexes against human miRNA-146a-5p were designed and synthesized by Gene Pharma Technologies (Shanghai, China). miRNA-146a-5p siRNA molecules were transfected individually into TCs using Lipofectamine RNAiMAX (Carlsbad, CA) according to the manufacturer’s instructions. Duplex siRNA was constructed against sequences coding for miRNA-146a-5p (5^′^-UGA GAA CUG AAU UCC AUG GGU U-3^′^). A scrambled negative control siRNA (5^′^-UUC UCC GAA CGU GUC ACG UTT-3^′^) was also included.

### Measurement of barrier permeability by transendothelial electrical resistance

The HPMEC monolayers’ transendothelial electrical resistance (TER) was measured with the electric cell-substrate impedance system (ECIS) as previously described [11]. The HPMECs were plated on gelatin-coated 8W10E electrode arrays at 1 × 10^5^  cells per well. The HPMECs were cultured until tight monolayers formed and stable TER values were reached. After the indicated stimulations to HPMEC monolayers, the changes in TER were recorded continuously by the ECIS system. The results were acquired at 4000 Hz.

### Statistical analysis

The data were analyzed with SPSS 17.0 statistics software (IBM Corporation, Endicott, NY, USA), and results are presented as mean ± standard deviation. Multiple-group comparisons were conducted using one-way analysis of variance, followed by Tukey’s *post hoc* test for multiple comparisons. An independent sample *t*-test was administered to compare the differences between two groups. *P* <.05 was considered statistically significant.

## Results

### TCs protect ECs from apoptosis under sepsis

**Figure 1:**
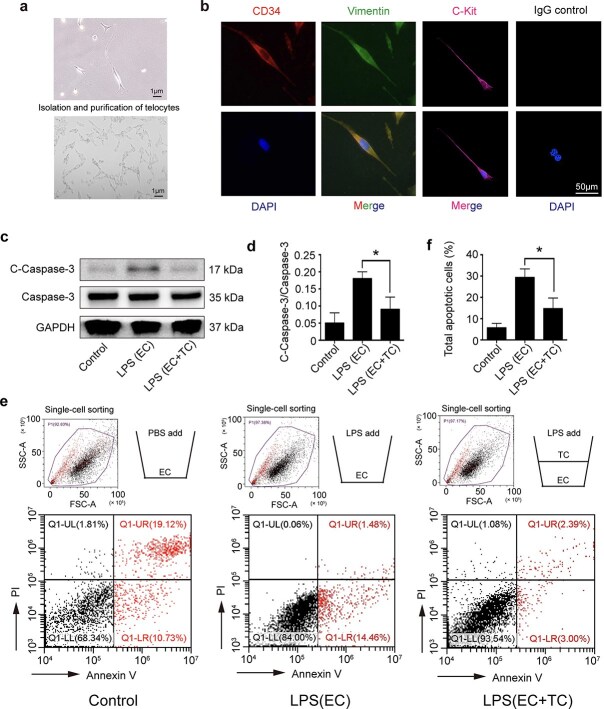
Isolation and purification of lung telocytes and their protective role to endothelial cells after LPS stimulation. (**a**) TCs with long telopodes under bright field. (**b**) Telocytes were identified by the immunofluorescence for CD34 (red), Vimentin (green), and C-kit (red), the non-antigen-specific IgG control is presented. (**c, d**) The cleaved caspase-3 expression and caspase-3 activation in ECs were detected by western blot after incubation with LPS (1 μg/ml, 24 h). (**e, f**) A transwell system was used to investigate the effect of TCs on ECs under septic conditions. There are three groups: the ECs + PBS (control) group, the ECs stimulated with LPS group, and a co-culture of ECs (in the lower chamber) with TCs (in the upper chamber) followed by LPS stimulation. For FACS of apoptosis cells, the total apoptosis rate is expressed as the sum of the populations in the upper right and lower right quadrants. ^*^P < .05. C-Caspase-3, cleaved caspase 3; TC, telocyte; LPS, lipopolysaccharide; EC, endothelial cell; GAPDH, glyceraldehyde-3-phosphatedehydrogenase

TCs were isolated from mouse lungs, and the typical long and thin telopodes of TCs were observed under a bright field (Figure 1a) [21]. TCs were positive for CD34, vimentin, and c-kit (Figure 1b) [22]. To explore the effects of TCs on ECs, we used transwell assays to simulate the relationships between TCs and ECs. ECs and TCs were cultured in the lower and upper chambers separately, and then, EC apoptosis was induced via LPS with or without TC co-culture. The experiment was divided into three groups: the control group (ECs + PBS), the LPS-treated group (ECs + LPS), and the TC-cocultured (ECs + TCs + LPS) group. LPS at a concentration of 1 μg/ml was added to the transwell assay when the cells were 70% confluent, and the control group was treated with PBS. Western blot analysis revealed that cleaved caspase-3 in ECs was significantly reduced when they were cocultured with TCs (Figure 1c, d), indicating that TCs protected ECs from LPS-induced apoptosis-associated proteins. Moreover, after incubation with LPS for 24 h, the number of apoptotic ECs was quantified via an Annexin V/PI apoptosis kit. We also found that the percentage of apoptotic ECs in the TC co-culture group was significantly lower than that in the LPS-treated group (Figure 1e, f). The evidence above suggests that under septic conditions, TC supernatants can inhibit the apoptosis of ECs.

### LPS increases the secretion of active TC exosomes

**Figure 2:**
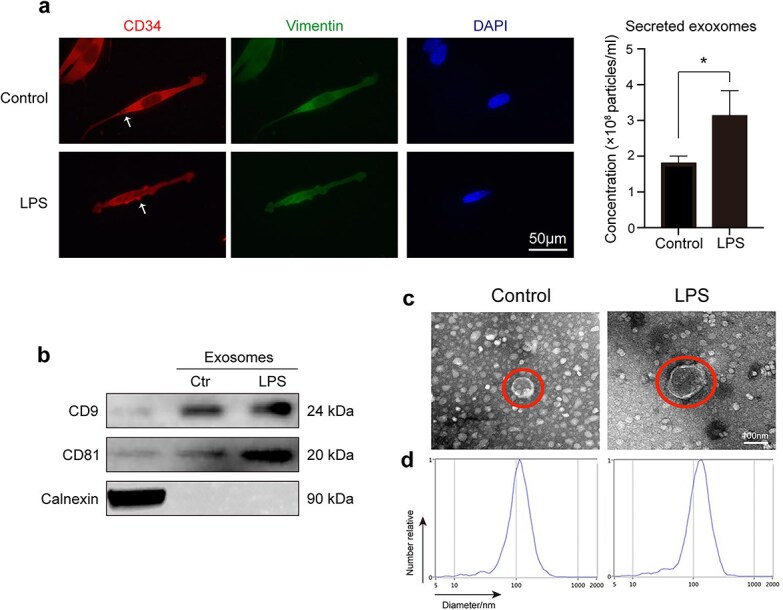
LPS-induced secretion of active TC exosomes may play a vital role on LPS-mediated EC injury. (**a**) Active TC exosomes are secreted from the intracellular to the extracellular area. The expulsion and volume of TC exosomes increased following LPS stimulation. White arrows indicate TC exosomes. (**b**) Western blot analysis was performed to identify TC exosomes by detecting proteins CD9, CD81, and Calnexin. (**c**) Transmission electron micrographs show that the diameter of active TC exosomes increased after stimulation of LPS. (**d**) Nanoparticle tracking analysis (NTA) was used to analyze the sizes of TC exosomes. ^*^P < .05. LPS, lipopolysaccharide; ctr, control

Cell supernatants contain different substances, including cell factors, proteins, and exosomes. Studies have shown that TCs can secrete exosomes that mediate their communication functions [17, 23]. To verify whether lung TCs protect ECs via exosomes during sepsis, we stimulated TCs with LPS and then observed the TC morphological changes via immunofluorescence. Compared with the control group, the LPS-treated TC group presented increases in both the volume and secretion of TC exosomes (Figure 2a). The exosomes were further characterized by detecting the exosomal marker proteins CD9, CD81, and Calnexin (Figure 2b). Moreover, an increased diameter of the exosomes was observed via transmission electron microscopy and nanoparticle tracking analysis (Figure 2c, d). These results demonstrated that LPS stimulation led to the regeneration and secretion of active TC exosomes.

### LPS-incubated active TC exosomes alleviate LPS-induced acute lung injury *in vivo*

**Figure 3:**
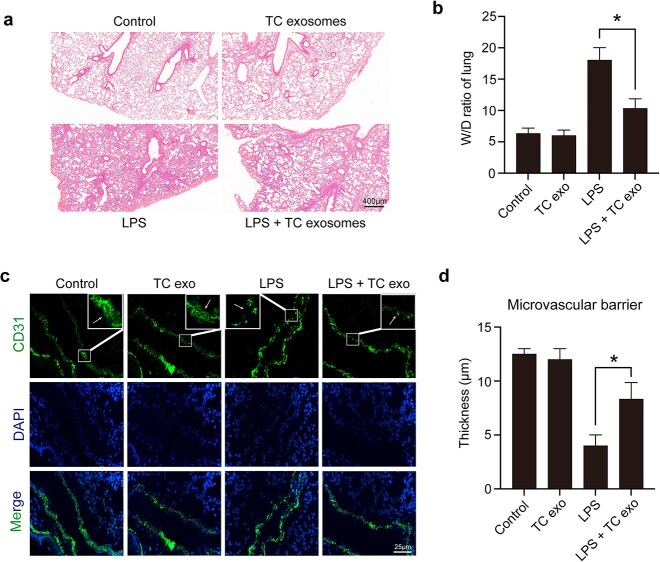
LPS-activated TC exosomes alleviated acute lung injury under septic conditions. (**a, b**) Assessing the general changes in mice lungs under different conditions by hematoxylin and eosin. LPS causes lung endothelial barrier disruption, inflammatory cell infiltration, and lung edema, which were obviously alleviated by LPS-activated TC exosomes (magnification, ×200). (**c, d**) Evaluating the changes in microvasculature by positive staining for CD31 (green) in lung tissue under different conditions. The lung microvascular barrier was seriously disrupted by LPS, but the vascular damage was reversed by LPS-induced active TC exosomes (magnification, ×400). ^*^P < .05. TC, telocyte; LPS, lipopolysaccharide; TC exo, telocyte exosomes; W/D, wet/dry

To clarify the protective role of LPS-activated TC exosomes in ALI during sepsis, we constructed a septic ALI model using LPS tracheal drops. We divided the mice into four groups: the control group, the LPS-induced active TC exosome group, the LPS-only group, and the LPS + LPS-activated TC exosome group. We detected changes in the lungs of the different groups via H&E staining, and the results revealed that LPS induced obvious ALI, such as endothelial wall rupture, alveolar cavity fusion, residual alveolar wall thickening, lung tissue edema, red blood cell appearance in the alveoli, and monocyte infiltration in the alveolar wall, in the mice. When septic mice were treated with LPS-activated TC exosomes, these manifestations of lung injury were considerably alleviated (Figure 3a). The W/D ratio indicated that lung edema was significantly alleviated with TC exosomes treatment compared to the LPS group (Figure 3b). Using immunofluorescence, we also observed that the integrity of the mouse lung microvasculature was disrupted by LPS; moreover, the injection of LPS-stimulated active TC exosomes effectively inhibited LPS-induced lung vascular barrier disruption and hyperpermeability (Figure 3c, d).

### LPS-stimulated active TC exosomes suppress EC apoptosis via caspase-3 activation

**Figure 4:**
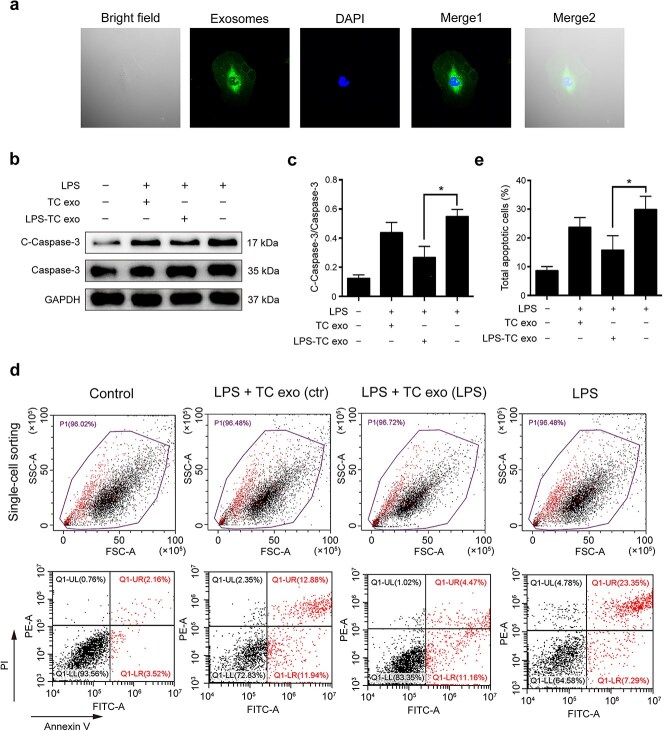
LPS-activated TC exosomes inhibit LPS-induced EC apoptosis. (**a**) The PKH67 (green) labeled TC exosomes were absorbed by ECs and were mainly located in cytoplasm of ECs. (**b, c**) LPS increases the expression of cleaved caspase-3 in ECs, and pretreatment with LPS-activated TC exosomes effectively inhibits LPS-induced caspase-3 activation. Compared with the LPS-activated TC exosomes, the normal TC exosomes have only a mild inhibitory effect on LPS-induced cleaved caspase-3 expression, and this effect is not statistically significant. (**d, e**) Pretreatment with LPS-activated TC exosomes effectively inhibits LPS-induced EC apoptosis. Compared to the LPS-activated TC exosomes, the normal TC exosomes have only a mild inhibitory effect on LPS-induced EC apoptosis, and this effect is not statistically significant. ^*^P < .05. LPS, lipopolysaccharide; TC, telocyte; TC exo, telocyte exosomes; LPS-TC exo, lipopolysaccharide-activated telocyte exosomes; C-Caspase-3, cleaved caspase-3; con, control

To explore whether LPS-activated TC exosomes are key protective molecules against ECs, LPS-stimulated active TC exosomes were extracted and labeled with PKH67 (green fluorescence). We found that TC exosomes were rapidly absorbed by ECs and were distributed mainly in the cytoplasm (Figure 4a). To investigate the differences between normal TC exosomes and LPS-activated TC exosomes, we divided the ECs into four groups: the control group, the LPS + normal TC exosome (TC treated with PBS) group, the LPS + LPS-activated TC exosome (TC treated with LPS) group, and the LPS group. The expression levels of cleaved caspase-3 and total caspase-3 were detected via western blotting. The results revealed that cleaved caspase-3 was significantly reduced upon the addition of LPS-activated TC exosomes (Figure 4b). Interestingly, although normal TC exosomes also inhibited LPS-induced cleaved caspase-3 expression in ECs to some extent, these effects were not statistically significant (Figure 4c). These data suggest that LPS-stimulated active TC exosomes play an important role in protection against LPS-induced EC apoptosis. The effect of TC exosomes on ECs was further confirmed via fluorescence-activated cell sorting (FACS): the percentage of apoptotic ECs in the LPS + LPS-activated TC exosome group was significantly lower than that in the LPS group, which revealed that LPS-activated TC exosomes effectively blocked LPS-induced EC apoptosis (Figure 4d, e).

### miR-146a-5p is the main protective molecule in LPS-stimulated active TC exosomes against LPS-induced EC apoptosis

**Figure 5:**
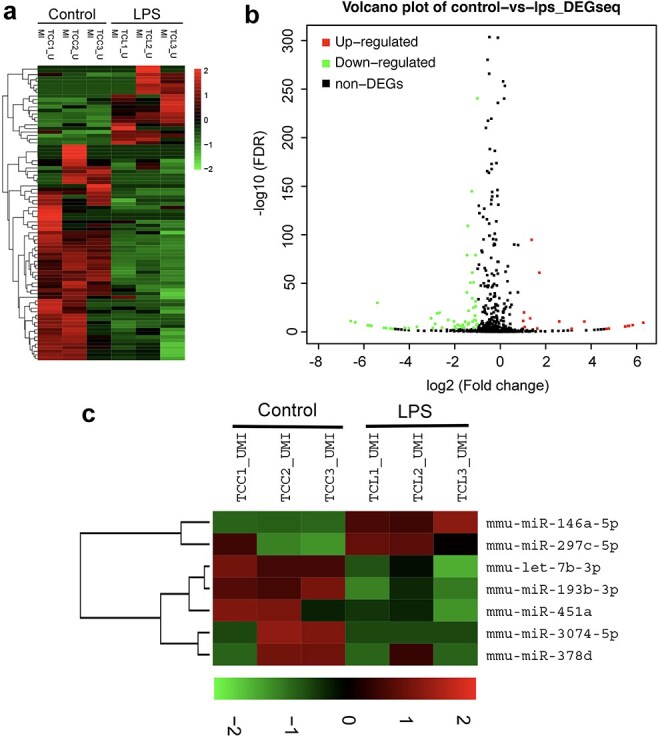
Analysis of small RNA sequencing of LPS-activated TC exosomes. (**a**) Heatmap showing the total differential expression of small RNAs in LPS-activated TC exosomes compared to normal TC exosomes (incubating with PBS). (**b**) Volcano plot illustrates the overall changes of miRNAs expression in LPS-activated TC exosomes. (**c**) miRNA-146a-5p was upregulated obviously in LPS-activated TC exosomes by the heatmap analysis. LPS lipopolysaccharide, miRNA micro RNA

**Figure 6:**
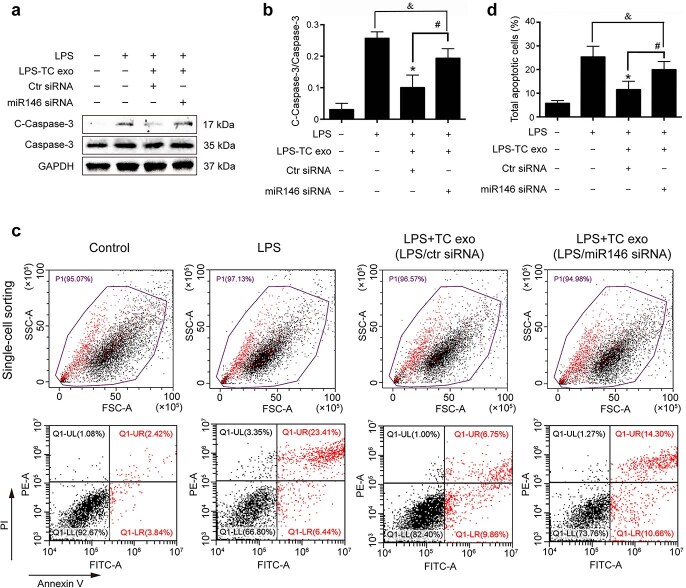
LPS-activated TC exosomes protect ECs from LPS-induced apoptosis via miRNA-146a-5p. (a, b) LPS increased the expression of cleaved caspase-3 in ECs, and pretreatment with LPS-activated TC exosomes effectively inhibited LPS-induced caspase-3 activation. However, when the expression of miRNA-146a-5p in TCs was blocked, the LPS-activated TC exosomes had only a mild inhibitory effect on LPS-induced cleaved caspase-3 expression, and this effect is not statistically significant. (c, d) Pretreatment with LPS-activated TC exosomes effectively inhibited LPS-induced EC apoptosis. However, when the expression of miRNA-146a-5p in TCs was blocked, the LPS-activated TC exosomes had only a mild inhibitory effect on LPS-induced EC apoptosis, and this effect is not statistically significant. ^*^P < .05, ^#^*P* < .05, ^&^no significance. LPS, lipopolysaccharide; TC, telocyte; TC exo, telocyte exosomes; LPS-TC exo, lipopolysaccharide-activated telocyte exosomes; miR146, microRNA 146a-5p; siRNA, small interfering RNA; C-Caspase-3, cleaved caspase-3

Exosomes carry receptors, bioactive lipids, proteins, and, most importantly, nucleic acids, such as mRNAs, microRNAs (miRNAs), and noncoding RNAs [23]. To identify the key functional molecules that perform the primary protective function of LPS-activated TC exosomes, the miRNA profile of TC exosomes with or without LPS stimulation was investigated via a small RNA sequencing assay. In total, 82 microRNAs were differentially expressed, with 22 upregulated and 60 downregulated, all exhibiting absolute fold changes >1.5 in the TCs after stimulation with LPS (Figure 5a, b). Notably, miRNA-146a-5p expression in the LPS-activated TC exosomes was significantly greater than that in the normal TC exosomes from the control group (Figure 5c). miRNA-146a-5p has been found to play an important role in many inflammatory processes, such as osteoarthritis [24] and bacterial sepsis [25]. To further evaluate whether miRNA-146a-5p in LPS-activated TC exosomes plays a key protective role in LPS-induced EC caspase-3 activation and apoptosis, we first used an siRNA assay to interfere with the expression of miRNA-146a-5p in LPS-stimulated TCs and then extracted exosomes from active TCs to costimulate ECs with LPS. Compared with that in the LPS group, the expression level of cleaved caspase-3 in ECs was significantly lower in the LPS + LPS-activated TC exosome (with miRNA-146a-5p) group. However, after the inhibition of miRNA-146a-5p expression in the LPS-activated TC exosomes, the protective effects of the activated TC exosomes on LPS-induced EC apoptosis and cleaved caspase-3 expression were significantly diminished. Furthermore, the levels of cleaved caspase-3 activation in the LPS + LPS-activated TC exosome (without miRNA-146a-5p) group were similar to those in the LPS-only group (Figure 6a, b). These results were further examined via FACS, which revealed that the LPS + LPS-incubated active TC exosome (containing miRNA-146a-5p) clearly blocked LPS-induced EC apoptosis. However, the inhibition of miRNA-146a-5p expression in LPS-stimulated active TC exosomes (LPS + LPS-activated TC exosomes) reversed the protective effect of these exosomes in preventing LPS-induced EC apoptosis (Figure 6c, d).

### miR-146a-5p in LPS-stimulated active TC exosomes plays an important role in LPS-induced EC barrier disruption

**Figure 7:**
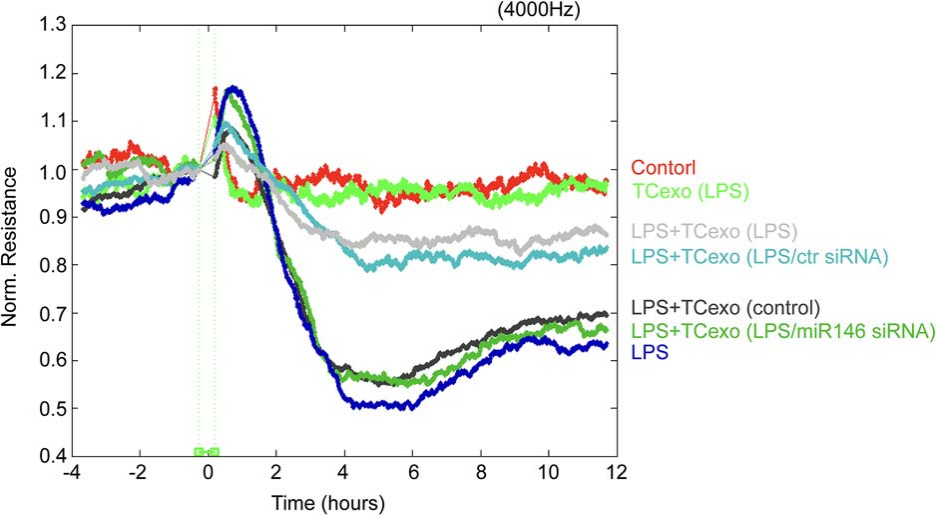
LPS-activated TC exosomes inhibit LPS-mediated ECs barrier disruption by miRNA-146a-5p. ECs were plated on the gold microelectrodes. When ECs formed the monolayer and the TER values were stable, different stimulations were added (control group, LPS-activated TCs exosomes group, LPS + LPS-activated TC exosomes, LPS group, LPS + normal TC exosomes, LPS + LPS-activated TC exosomes without miRNA- 146a-5p). the ECs monolayer permeability was assessed by real-time TER measurements. LPS lipopolysaccharide, TC telocyte, TER trans-endothelial electrical resistance, ECIS electrical cell-substrate impedance sensing, miR-146 micro RNA-146a-5p, siRNA small interfering RNA

To further investigate the underlying effects of TC exosomes on the integrity of the EC barrier after stimulation with LPS, we divided ECs into seven groups: (1) the PBS group (control), (2) the LPS group, (3) the LPS-activated TC exosome group, (4) the LPS + TC exosome group, (5) the LPS + LPS-activated TC exosome group, (6) the LPS + LPS-activated TC exosome with control siRNA group, and (7) the LPS + LPS-activated TC exosome with miRNA-146a-5p siRNA group. The permeability of ECs was measured by TER. Consistent with previous studies [11], LPS rapidly induced a decrease in TER and barrier disruption within 4–6 h. Moreover, the TER of the LPS + TC exosome (without LPS stimulation) group was comparable to that of the LPS group, indicating that LPS-activated TC exosomes are crucial for protecting against LPS-induced EC hyperpermeability, whereas normal TC exosomes are not under physiological conditions. Moreover, we observed that the LPS + LPS-activated TC exosome with control siRNA group obviously inhibited LPS-induced EC barrier disruption, but the LPS + LPS-activated TC exosome with miRNA-146a-5p siRNA group reduced the protective effect of the LPS-activated TC exosomes. These findings suggest that miRNA-146a-5p is a key molecule in LPS-activated TC exosomes that prevents LPS-induced EC barrier disruption (Figure 7). These results indicate that miRNA-146a-5p, a key functional molecule in LPS-activated TC exosomes, effectively blocks LPS-induced EC apoptosis and barrier disruption by modulating the miRNA-146a-5p/caspase-3 signaling pathway.

## Discussion

Lipopolysaccharide (LPS)-induced apoptosis of microvascular ECs damages the integrity of the alveolar–capillary membrane and induces pulmonary edema, which are the main causes of ALI under septic conditions [3–5]. Although many previous studies have revealed new anti-apoptosis drugs for sepsis, these drugs are not always effective at protecting ECs under septic conditions [26, 27]. TCs are cells identified in the interstitial space of many organs, including the lungs, that protect adjacent cells and participate in tissue repair and regeneration [13, 21, 28]. TCs are found around pulmonary microvascular endothelial cells, but whether TCs protect against LPS-induced EC apoptosis and vascular barrier disruption is not clear [22, 29]. In this study, we demonstrated that under septic conditions, TCs can secrete many active exosomes to block LPS-induced EC apoptosis and vascular barrier disruption and subsequently alleviate LPS-induced ALI. We further revealed that miRNA-146a-5p is the main effective molecule in LPS-stimulated active TC exosomes and that it suppresses caspase-3 activation and apoptosis in ECs during sepsis.

Telocytes are cells with long and thin prolongations that have been identified in the interstitial space of many organs [13, 28, 30]. TCs protect adjacent cells and participate in tissue repair and regeneration. A recent study reported that cardiac TC can inhibit the apoptosis of ECs under ischemia–hypoxia conditions [31]. To explore whether lung TCs protect against LPS-induced EC apoptosis and microvascular barrier disruption, we first extracted primary TCs from mouse lungs and then cocultured ECs and TCs with LPS in a transwell assay to simulate the interaction between both cell types under septic conditions. The results indicated that LPS-stimulated TC supernatant significantly reduced LPS-induced caspase-3 activation and apoptosis in ECs. To our knowledge, these data demonstrate for the first time that lung TCs can significantly reduce pulmonary microvascular endothelial cell apoptosis during sepsis.

TCs mainly affect surrounding cells by secreting exosomes [17, 18, 31, 32]. An increasing number of researchers have reported that exosomes play an important role in restoring vascular endothelial cell injury [33–36]. Therefore, we further extracted exosomes from TCs and evaluated exosome function with or without LPS stimulation. We found that LPS increased the volume and secretion of TC exosomes, which may contain more active molecules and accelerate the recovery of TCs under pathological conditions. These results were in accordance with the data of the above transwell experiments, which indicated that TCs might be activated and secrete active exosomes to protect against LPS-induced EC apoptosis during sepsis. To explore whether LPS-induced active TC exosomes play a protective role in LPS-induced EC barrier disruption and ALI, we extracted active exosomes from LPS-stimulated TCs and then injected them into septic mice. We found that LPS-induced active TC exosomes obviously reversed LPS-induced pulmonary microvascular endothelial barrier disruption and effectively blocked LPS-induced lung edema and ALI.

To further determine the underlying protective mechanism of LPS-induced active TC exosomes, we tested their function *in vitro*. We found that LPS-induced active TC exosomes were absorbed by ECs and were distributed mainly in the EC cytoplasm. Moreover, we found that LPS-induced active TC exosomes significantly inhibited LPS-induced caspase-3 activation and apoptosis in ECs, which is consistent with the results of the above tests *in vivo*. Interestingly, we also found that normal TC exosomes without LPS stimulation had a less protective effect on LPS-induced caspase-3 activation and apoptosis in ECs. Hence, the above data suggest that TCs in a physiological state have a mild protective effect on surrounding ECs; however, under pathological conditions, such as septic conditions, LPS-induced active TC exosomes have a stronger protective effect on LPS-induced EC injury. These results provide the first evidence that TCs play an obviously protective role on ECs via exosomes during sepsis.

Exosomes contain many molecules, such as nucleic acids, proteins, amino acids, and metabolites. Cells conduct genetic information exchange and cell reprogramming through exocrine systems [37, 38]. As posttranscriptional regulators, miRNAs are closely associated with infection and sepsis progression [39]. Host cellular RNAs are released into the blood during certain critical illnesses and perform multiple functions. For example, exosomal miRNA-21-5p targets and silences the death-inducing p53 target 1 gene and thus downregulates activated caspase-3, which then inhibits the apoptosis of recipient endothelial cells under ischemic and hypoxic conditions [31]. To gain further insight into the role of miRNAs in LPS-stimulated active TC exosomes, we investigated the changes in total miRNA levels in TC exosomes with or without LPS stimulation via RNA sequencing. The results demonstrated that the expression of miRNA-146 was obviously increased in the LPS-stimulated active TC exosome group. However, whether LPS-induced active TC exosomes protect against LPS-induced EC injury mainly through miRNA-146 is unknown. In the present study, we further found that inhibiting the expression of miRNA-146 in LPS-induced active TC exosomes effectively inhibited the protective effect of LPS-induced active TC exosomes on LPS-induced EC apoptosis. These results demonstrated that miRNA-146 plays an important role in the anti-apoptotic effect of LPS-stimulated active TC exosomes on ECs during sepsis. The intrinsic apoptotic signaling pathway is regulated mainly by caspase family proteins that play a key role in LPS-induced pulmonary EC apoptosis [4]. Here, we further explored the role of LPS-stimulated active TC exosomes with or without miRNA-164 in the activation of caspase-3 in LPS-incubated ECs. We found that LPS-stimulated active TC exosomes effectively inhibited LPS-induced caspase-3 activation and cleaved caspase-3 in ECs, but blocking the expression of miRNA-146 in LPS-stimulated active TC exosomes clearly reversed the protective effect of LPS-stimulated active TC exosomes on EC apoptosis during sepsis.

Lung vascular EC apoptosis is the main reason for the disruption of the lung microvascular barrier [40, 41]; however, whether LPS-stimulated active TC exosomes containing miRNA-146 are involved in the regulation of EC monolayer barrier function during sepsis remains poorly defined. In this study, the results demonstrated that LPS-incubated active TC exosomes effectively inhibited LPS-induced disruption of the EC monolayer barrier, but inhibiting the expression of miRNA-146 in LPS-stimulated active TC exosomes effectively reversed the protective effect of LPS-stimulated active TC exosomes on disruption of the EC monolayer barrier during sepsis. The above data indicated that TCs might inhibit LPS-induced EC apoptosis and block LPS-induced disruption of the EC monolayer barrier through the transmission of active exosomes from TCs to ECs and further activation of the miRNA-146/caspase-3 signaling pathway in ECs under septic conditions. All the above results revealed that, under physiological conditions, exosomes from TCs had only mild protective effects and could not reverse LPS-induced EC injury; however, under pathological conditions, such as septic conditions, LPS-induced active TC exosomes obviously protected against LPS-induced EC apoptosis and vascular barrier disruption via miRNA-146 transcellular transport and subsequent miRNA-194/caspase-3 signaling pathway activation in ECs.

## Conclusions

This study demonstrates that TCs protect against LPS-induced EC apoptosis and ALI by active exosomes. These active exosomes of TCs, which are synthesized and released by LPS stimulation, inhibit EC apoptosis through the miRNA-146a-5p/caspase-3 signaling pathway. Our findings suggest that TCs play a crucial role in maintaining lung microvascular integrity under septic conditions, offering a potential therapeutic approach for preventing lung injury in sepsis in the future.

## Data Availability

The datasets generated and analyzed during the current study are available from the corresponding author on reasonable request.

## References

[1] Font MD, Thyagarajan B, Khanna AK. Sepsis and septic shock - basics of diagnosis, pathophysiology and clinical decision making. *Med Clin North Am* 2020;104:573–85. 10.1016/j.mcna.2020.02.011. 32505253

[2] Liu D, Huang SY, Sun JH, Zhang HC, Cai QL, Gao C, et al. Sepsis-induced immunosuppression: mechanisms, diagnosis and current treatment options. *Mil Med Res* 2022;9:56. 10.1186/s40779-022-00422-y. 36209190 PMC9547753

[3] Guo F, Tang J, Zhou Z, Dou Y, Van Lonkhuyzen D, Gao C, et al. GEF-H1-RhoA signaling pathway mediates LPS-induced NF-kappaB transactivation and IL-8 synthesis in endothelial cells. *Mol Immunol* 2012;50:98–107. 10.1016/j.molimm.2011.12.009. 22226472

[4] Yi L, Huang X, Guo F, Zhou Z, Chang M, Tang J, et al. Lipopolysaccharide induces human pulmonary micro-vascular endothelial apoptosis via the YAP signaling pathway. *Front Cell Infect Microbiol* 2016;6:133. 10.3389/fcimb.2016.00133. 27807512 PMC5069405

[5] Hou X, Yang S, Yin J. Blocking the REDD1/TXNIP axis ameliorates LPS-induced vascular endothelial cell injury through repressing oxidative stress and apoptosis. *Am J Physiol Cell Physiol* 2019;316:C104–10. 10.1152/ajpcell.00313.2018. 30485138

[6] Guo J, Chen W, Bao B, Zhang D, Pan J, Zhang M. Protective effect of berberine against LPS-induced endothelial cell injury via the JNK signaling pathway and autophagic mechanisms. *Bioengineered* 2021;12:1324–37. 10.1080/21655979.2021.1915671. 33896366 PMC8806223

[7] Duan X, Yang Y, Yang A, Zhao Y, Fan F, Niu L, et al. Terbutaline attenuates LPS-induced injury of pulmonary microvascular endothelial cells by cAMP/Epac signaling. *Drug Dev Res* 2022;83:699–707. 10.1002/ddr.21901. 34846077

[8] Opal SM, van der Poll T. Endothelial barrier dysfunction in septic shock. *J Intern Med* 2015;277:277–93. 10.1111/joim.12331. 25418337

[9] Mu S, Liu Y, Jiang J, Ding R, Li X, Li X, et al. Unfractionated heparin ameliorates pulmonary microvascular endothelial barrier dysfunction via microtubule stabilization in acute lung injury. *Respir Res* 2018;19:220. 10.1186/s12931-018-0925-6. 30442128 PMC6238311

[10] Zhou Z, Guo F, Yi L, Tang J, Dou Y, Huan J. The p38/mitogen-activated protein kinase pathway is implicated in lipopolysaccharide-induced microtubule depolymerization via up-regulation of microtubule-associated protein 4 phosphorylation in human vascular endothelium. *Surgery* 2015;157:590–8. 10.1016/j.surg.2014.10.007. 25633728

[11] Yi L, Huang X, Guo F, Zhou Z, Chang M, Huan J. GSK-3Beta-dependent activation of GEF-H1/ROCK signaling promotes LPS-induced lung vascular endothelial barrier dysfunction and acute lung injury. *Front Cell Infect Microbiol* 2017;7:357. 10.3389/fcimb.2017.00357. 28824887 PMC5543036

[12] Dutra Silva J, Su Y, Calfee CS, Delucchi KL, Weiss D, McAuley DF, et al. Mesenchymal stromal cell extracellular vesicles rescue mitochondrial dysfunction and improve barrier integrity in clinically relevant models of ARDS. *Eur Respir J* 2021;58:2002978. 10.1183/13993003.02978-2020. 33334945 PMC8318599

[13] Popescu LM, Gherghiceanu M, Suciu LC, Manole CG, Hinescu ME. Telocytes and putative stem cells in the lungs: electron microscopy, electron tomography and laser scanning microscopy. *Cell Tissue Res* 2011;345:391–403. 10.1007/s00441-011-1229-z. 21858462 PMC3168741

[14] Song D, Xu M, Qi R, Ma R, Zhou Y, Wu D, et al. Influence of gene modification in biological behaviors and responses of mouse lung telocytes to inflammation. *J Transl Med* 2019;17:158. 10.1186/s12967-019-1870-y. 31092264 PMC6521571

[15] Wollheim FA . Telocytes, communicators in healthy stroma and relation to inflammation and fibrosis. *Joint Bone Spine* 2016;83:615–8. 10.1016/j.jbspin.2016.06.002. 27452296

[16] Creţoiu SM . Telocytes and other interstitial cells: from structure to function. *Int J Mol Sci* 2021;22:5271. 10.3390/ijms22105271. PMC8156259 34067777

[17] Yang J, Li Y, Xue F, Liu W, Zhang S. Exosomes derived from cardiac telocytes exert positive effects on endothelial cells. *Am J Transl Res* 2017;9:5375–87. 29312490 PMC5752888

[18] Aschacher T, Aschacher O, Schmidt K, Enzmann FK, Eichmair E, Winkler B, et al. The role of telocytes and telocyte-derived exosomes in the development of thoracic aortic aneurysm. *Int J Mol Sci* 2022;23:4730. 10.3390/ijms23094730. PMC9099883 35563123

[19] Tang L, Song D, Qi R, Zhu B, Wang X. Roles of pulmonary telocytes in airway epithelia to benefit experimental acute lung injury through production of telocyte-driven mediators and exosomes. *Cell Biol Toxicol* 2023;39:451–65. 10.1007/s10565-021-09670-5. 34978009 PMC8720540

[20] Zhang C, Guo F, Chang M, Zhou Z, Yi L, Gao C, et al. Exosome-delivered syndecan-1 rescues acute lung injury via a FAK/p190RhoGAP/RhoA/ROCK/NF-κB signaling axis and glycocalyx enhancement. *Exp Cell Res* 2019;384:111596. 10.1016/j.yexcr.2019.111596. 31487506

[21] Popescu LM, Faussone-Pellegrini MS. TELOCYTES - a case of serendipity: the winding way from interstitial cells of Cajal (ICC), via interstitial Cajal-like cells (ICLC) to TELOCYTES. *J Cell Mol Med* 2010;14:729–40. 10.1111/j.1582-4934.2010.01059.x. 20367664 PMC3823108

[22] Cantarero I, Luesma MJ, Junquera C. The primary cilium of telocytes in the vasculature: electron microscope imaging. *J Cell Mol Med* 2011;15:2594–600. 10.1111/j.1582-4934.2011.01312.x. 21435170 PMC4373428

[23] Cretoiu D, Xu J, Xiao J, Cretoiu SM. Telocytes and their extracellular vesicles-evidence and hypotheses. *Int J Mol Sci* 2016;17:1322. 10.3390/ijms17081322. PMC5000719 27529228

[24] Li J, Huang J, Dai L, Yu D, Chen Q, Zhang X, et al. miR-146a, an IL-1beta responsive miRNA, induces vascular endothelial growth factor and chondrocyte apoptosis by targeting Smad4. *Arthritis Res Ther* 2012;14:R75. 10.1186/ar3798. 22507670 PMC3446449

[25] Wang S, Yang Y, Suen A, Zhu J, Williams B, Hu J, et al. Role of extracellular microRNA-146a-5p in host innate immunity and bacterial sepsis. *iScience* 2021;24:103441. 10.1016/j.isci.2021.103441. 34877498 PMC8633977

[26] Yi L, Chang M, Zhao Q, Zhou Z, Huang X, Guo F, et al. Genistein-3′-sodium sulphonate protects against lipopolysaccharide-induced lung vascular endothelial cell apoptosis and acute lung injury via BCL-2 signalling. *J Cell Mol Med* 2020;24:1022–35. 10.1111/jcmm.14815. 31756053 PMC6933390

[27] Yi L, Zhou Z, Zheng Y, Chang M, Huang X, Guo F, et al. Suppressive effects of GSS on lipopolysaccharide-induced endothelial cell injury and ALI via TNF-α and IL-6. *Mediat Inflamm* 2019;2019:1–13. 10.1155/2019/4251394. PMC7012263 32082076

[28] Bei Y, Zhou Q, Sun Q, Xiao J. Telocytes in cardiac regeneration and repair. *Semin Cell Dev Biol* 2016;55:14–21. 10.1016/j.semcdb.2016.01.037. 26826525

[29] Zheng Y, Li H, Manole CG, Sun A, Ge J, Wang X. Telocytes in trachea and lungs. *J Cell Mol Med* 2011;15:2262–8. 10.1111/j.1582-4934.2011.01404.x. 21810171 PMC4394233

[30] Kondo A, Kaestner KH. Emerging diverse roles of telocytes. *Development* 2019;146:146. 10.1242/dev.175018. PMC6679369 31311805

[31] Liao Z, Chen Y, Duan C, Zhu K, Huang R, Zhao H, et al. Cardiac telocytes inhibit cardiac microvascular endothelial cell apoptosis through exosomal miRNA-21-5p-targeted cdip1 silencing to improve angiogenesis following myocardial infarction. *Theranostics* 2021;11:268–91. 10.7150/thno.47021. 33391474 PMC7681094

[32] Zhou Y, Yang Y, Liang T, Hu Y, Tang H, Song D, et al. The regulatory effect of microRNA-21a-3p on the promotion of telocyte angiogenesis mediated by PI3K (p110α)/AKT/mTOR in LPS induced mice ARDS. *J Transl Med* 2019;17:427. 10.1186/s12967-019-02168-z. 31878977 PMC6933909

[33] Mizuta Y, Akahoshi T, Guo J, Zhang S, Narahara S, Kawano T, et al. Exosomes from adipose tissue-derived mesenchymal stem cells ameliorate histone-induced acute lung injury by activating the PI3K/Akt pathway in endothelial cells. *Stem Cell Res Ther* 2020;11:508. 10.1186/s13287-020-02015-9. 33246503 PMC7691956

[34] Yu Q, Wang D, Wen X, Tang X, Qi D, He J, et al. Adipose-derived exosomes protect the pulmonary endothelial barrier in ventilator-induced lung injury by inhibiting the TRPV4/Ca(2+) signaling pathway. *Am J Physiol Lung Cell Mol Physiol* 2020;318:L723–41. 10.1152/ajplung.00255.2019. 32073873 PMC7191475

[35] Xu J, Xu D, Yu Z, Fu Z, Lv Z, Meng L, et al. Exosomal miR-150 partially attenuated acute lung injury by mediating microvascular endothelial cells and MAPK pathway. *Biosci Rep* 2022;42:BSR20203363. 10.1042/BSR20203363. PMC8703023 34750610

[36] Shen K, Wang X, Wang Y, Jia Y, Zhang Y, Wang K, et al. miR-125b-5p in adipose derived stem cells exosome alleviates pulmonary microvascular endothelial cells ferroptosis via Keap1/Nrf2/GPX4 in sepsis lung injury. *Redox Biol* 2023;62:102655. 10.1016/j.redox.2023.102655. 36913799 PMC10023991

[37] Qin J, Xu Q. Functions and application of exosomes. *Acta Pol Pharm* 2014;71:537–43. 25272880

[38] Kalluri R, LeBleu VS. The biology, function, and biomedical applications of exosomes. *Science* 2020;367:eaau6977. 10.1126/science.aau6977. PMC7717626 32029601

[39] Fabri-Faja N, Calvo-Lozano O, Dey P, Terborg RA, Estevez MC, Belushkin A, et al. Early sepsis diagnosis via protein and miRNA biomarkers using a novel point-of-care photonic biosensor. *Anal Chim Acta* 2019;1077:232–42. 10.1016/j.aca.2019.05.038. 31307714

[40] Wang Z, Guo Z, Wang X, Liao H, Chen F, Liu Y, et al. Reduning alleviates sepsis-induced acute lung injury by reducing apoptosis of pulmonary microvascular endothelial cells. *Front Immunol* 2023;14:1196350. 10.3389/fimmu.2023.1196350. 37465664 PMC10350519

[41] Simmons S, Erfinanda L, Bartz C, Kuebler WM. Novel mechanisms regulating endothelial barrier function in the pulmonary microcirculation. *J Physiol* 2019;597:997–1021. 10.1113/JP276245. 30015354 PMC6375872

